# Short-Term Effects of Climate Variability on Childhood Diarrhoea in Bangladesh: Multi-Site Time-Series Regression Analysis

**DOI:** 10.3390/ijerph20136279

**Published:** 2023-07-02

**Authors:** Md Rezanur Rahaman, Keith Dear, Syed M. Satter, Michael Tong, Adriana Milazzo, Helen Marshall, Blesson M. Varghese, Mahmudur Rahman, Peng Bi

**Affiliations:** 1 National Centre for Epidemiology and Population Health, The Australian National University, Canberra, ACT 2601, Australia; 2 School of Public Health, The University of Adelaide, Adelaide, SA 5005, Australia; 3 International Centre for Diarrhoeal Disease Research, Bangladesh (icddr,b), Dhaka 1212, Bangladesh; 4 Adelaide Medical School and Robinson Research Institute, The University of Adelaide, Adelaide, SA 5005, Australia; 5 Women’s and Children’s Health Network, Adelaide, SA 5006, Australia

**Keywords:** Bangladesh, children, climate, diarrhoea, maximum temperature, time-series

## Abstract

The aim of this study was to estimate the effects of climate on childhood diarrhoea hospitalisations across six administrative divisions in Bangladesh and to provide scientific evidence for local health authorities for disease control and prevention. Fortnightly hospital admissions (August/2013–June/2017) for diarrhoea in children under five years of age, and fortnightly average maximum temperature, relative humidity and rainfall recordings for six administrative divisions were modelled using negative binomial regression with distributed lag linear terms. Flexible spline functions were used to adjust models for seasonality and long-term trends. During the study period, 25,385 diarrhoea cases were hospitalised. Overall, each 1 °C rise in maximum temperature increased diarrhoea hospitalisations by 4.6% (IRR = 1.046; 95% CI, 1.007–1.088) after adjusting for seasonality and long-term trends in the unlagged model. Using lagged effects of maximum temperature, and adjusting for relative humidity and rainfall for each of the six administrative divisions, the relationship between maximum temperature and diarrhoea hospitalisations varied between divisions, with positive and negative effect estimates. The temperature-diarrhoea association may be confounded by seasonality and long-term trends. Our findings are a reminder that the effects of climate change may be heterogeneous across regions, and that tailored diarrhoea prevention strategies need to consider region-specific recommendations rather than relying on generic guidelines.

## 1. Introduction

Diarrhoea is ranked the fifth cause of global deaths among children under five years of age [1]. Globally, over half a million children under five years of age die each year from diarrhoea [2]. South Asia and sub-Saharan Africa bear 90% of the global burden of diarrhoeal deaths [3]. Not surprisingly, malnutrition, inadequate sanitation and unsafe drinking water are major health issues in both of these regions, and are the risk factors for diarrhoea for the majority of these children identified by the Global Burden of Disease Study [1]. Identified major diarrhoeal pathogens among children under five years old in these regions include rotavirus, *Shigella*, norovirus and adenovirus [4]. The World Health Organization has estimated that improved sanitation and safe water supply alone can avert the majority of these diarrhoeal diseases [2]. Despite success in the South Asia region in reducing diarrhoeal deaths [5], Bangladesh remains highly vulnerable to diarrhoeal diseases [6]. Such risks could potentially be linked to a geographically diverse climate differentially affecting a dense population [7]. Despite significant improvements in hygiene and diarrhoea management at a population level, an increasing number of diarrhoeal epidemics each year in Bangladesh have placed the country in a unique position to examine diarrhoea incidence and climate factors in an effort to reduce childhood diarrhoea hospitalisations [3,8].

Global preventable deaths from diarrhoea among children under five years old are projected to fall below 1 death per 1000 live births by 2025 [9]. In order to help attain this projection, countries need to scale up existing interventions, including integrated management of illnesses at hospitals, integrated case management in the community, childhood immunization and community health education campaigns to reduce the risk factors [9]. In the face of climate change and global warming, extreme weather events including cyclones and floods are a common occurrence in Bangladesh [8]. These events often have enduring impacts on people’s health and living, with vulnerable populations, particularly children, bearing a disproportionate burden of infectious diseases, including diarrhoea [10]. Evidence suggests that scaling up existing diarrhoea prevention interventions in Bangladesh could be supplemented by modelling climate variables and generating findings that translate into public health policy and practice to avert excess childhood morbidity and mortality with better predictability and preparedness [8].

Evidence suggests that analysis of systematically collected, nationally representative surveillance data may provide deeper insights into the epidemiology of childhood diarrhoea and help formulate country-specific preventative strategies [4]. We aimed to delineate how climate conditions such as maximum temperature, relative humidity and rainfall may influence the incidence of childhood diarrhoea hospitalisations in Bangladesh, in order to inform diarrhoea prevention policies leading to regionally tailored prevention strategies. The primary aim was to investigate whether the variation in the number of childhood diarrhoea hospitalisations (outcome) can be explained in part by changes in maximum temperature (main exposure), overall and across each of the six administrative divisions, and so to provide scientific evidence from which local health authorities might develop appropriate public health policy for practice.

## 2. Materials and Methods

### 2.1. Study Setting

Bangladesh is located in Southern Asia, bordering the Bay of Bengal, between Myanmar and India. In a total area of 148,460 square kilometres, the country is home to approximately 170 million people, including 16.3 million (9.6%) children under five years of age [11]. Comprising 88% land, 12% water and 580 kilometres of coastline, Bangladesh suffers from droughts, cyclones and frequent inundation, particularly during the monsoon season [12]. Flooding is mostly due to the country’s geography, as most of it is situated on deltas of large rivers such as the Ganges and Brahmaputra, which flow from the Himalayas and end in the Bay of Bengal. Bangladesh is a tropical country with a relatively mild winter, but with a very hot and humid summer and a humid rainy season. A flat terrain, proximity to the Indus-Ganges-Brahmaputra Basin aquifers, large agricultural land use with reliance on river and rainwater irrigation, frequent post-monsoon inundation and periodic cyclones contribute to the country’s periodic natural disasters [12]. The country’s tropical climate favours the transmission and incidence of infectious diseases, particularly diarrhoea, which is frequently compounded by the occurrence of periodic natural disasters [12].

### 2.2. Surveillance Hospitals and Weather Stations

**Figure 1:**
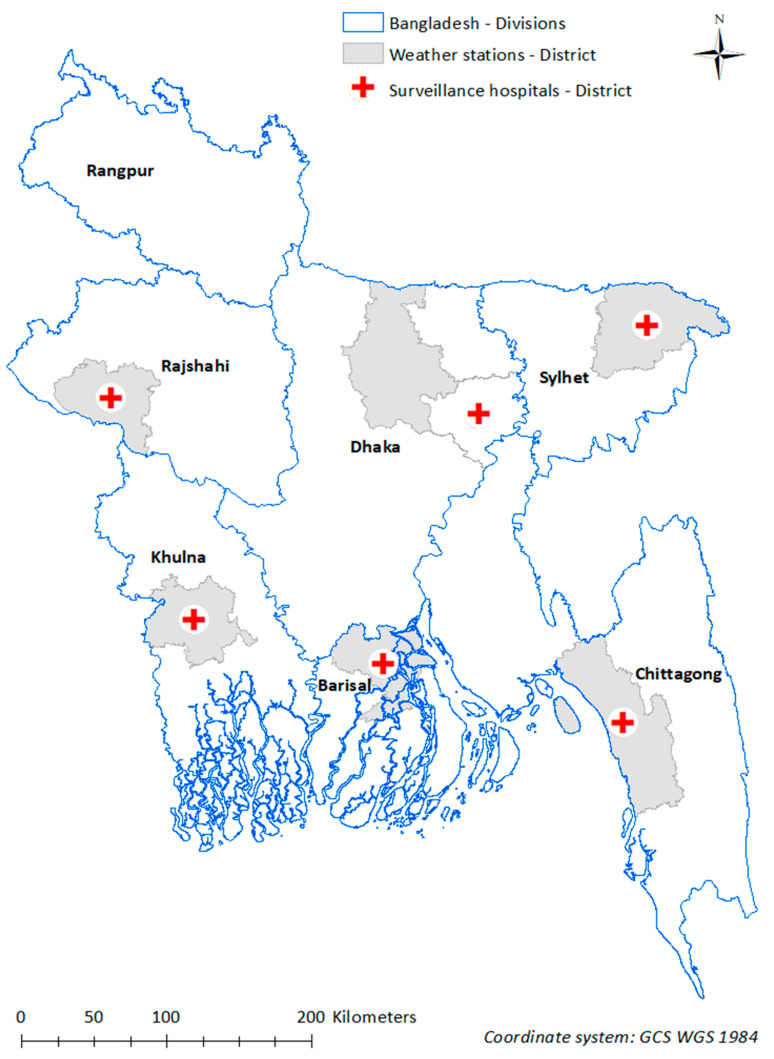
Location of diarrhoea surveillance hospitals and corresponding weather stations. The administrative divisions, locations for the study sites, are Barisal, Chittagong, Dhaka, Khulna, Rajshahi and Sylhet.

Bangladesh consists of seven administrative divisions [13], each comprising several districts within the administrative level. The seven administrative divisions are Barisal, Chittagong, Dhaka, Khulna, Rajshahi, Rangpur and Sylhet―only six, except Rangpur, are included in this current study (Figure 1). There are six tertiary surveillance hospitals, one in each administrative division, with the exception of Rangpur. Except for Dhaka, these hospitals are located in the most populous district of each administrative division. Weather stations are located in the same districts as the surveillance hospitals, except for Dhaka, the weather station of which is in the neighbouring district (Figure 1).

### 2.3. Diarrhoea Surveillance

Diarrhoea surveillance was conducted among children under five years of age in six tertiary teaching hospitals across Bangladesh with their division-wide catchments (Figure 1). Diarrhoea surveillance in Bangladesh started in 2012 with the broader aims of monitoring the trends of childhood diarrhoea incidence, examining age, geographical region and seasonal distribution of diarrhoea-related hospitalisations, and estimating the frequency of complications associated with childhood diarrhoea hospitalisations [14]. For the purpose of this surveillance, acute diarrhoea was defined as ≥3 loose stools per day with symptoms lasting for ≤7 days [14]. The study outcome was fortnightly counts of children under five years hospitalised with diarrhoea in each of the six surveillance hospitals from August 2013 to June 2017. The diarrhoea surveillance team comprised physicians responsible for epidemiological data collection, nurses coordinating with physicians and the laboratory and laboratory technicians responsible for stool specimen collection. Although surveillance staff screened and enrolled eligible children and collected stool samples daily, specimens were fortnightly sent to the laboratory at the International Centre for Diarrhoeal Disease Research, Bangladesh (icddr,b) for testing [15]. Hence, we used fortnightly diarrhoea count as the outcome. The Institute of Epidemiology, Disease Control and Research (a national partner) provides support to the surveillance system with technical assistance being provided by the U.S. Centres for Disease Control and Prevention, the USAID and the World Health Organization.

### 2.4. Meteorological Data

We obtained daily maximum temperature (T-max), relative humidity (RH) and total rainfall (rainfall) data from the Bangladesh Meteorological Department for each of the six administrative divisions (study sites), as shown in Figure 1. Weather data were representative of the whole administrative division and not just the district containing the weather station. We calculated the mean of daily maximum temperature, relative humidity and rainfall to generate fortnightly weather recordings in order to keep the time intervals consistent with diarrhoea counts over the study period.

### 2.5. Descriptive Analysis

We examined the data using summary statistics, frequency tables, correlation matrices and scatter plots. Systematic patterns such as seasonality in the data were examined using scatter plots for fortnightly maximum temperature, relative humidity and rainfall recordings (exposure) and fortnightly diarrhoea counts (outcome) over the study period.

### 2.6. Time-Series Negative Binomial Regression

We applied a time-series negative binomial regression (NBR) model to account for overdispersion in the data [16,17]. In addition, an NBR allows for controlling multiple potential confounders, such as relative humidity and rainfall. However, the assumption of independence was violated as observations were autocorrelated, with temporally proximal observations being more similar than temporally distant ones. This autocorrelation may not be inherent to counts of diarrhoea, but could be due to autocorrelation in the exposure variables [18]. Autocorrelation appears to be more common for infectious diseases than non-infectious diseases [16]. We dealt with autocorrelation by controlling for seasonality and long-term trends, as well as through model checking and sensitivity analysis, as suggested by recent evidence [18].

### 2.7. Seasonality and Long-Term Trends

As our dataset was dominated by seasonality and long-term trends, we controlled for this using flexible spline functions. Although flexibility can be achieved by a high number of degrees of freedom per year, i.e., more degrees of freedom allow more flexibility, a balance is recommended to avoid failure to capture long-term patterns (too few degrees of freedom) and not to overfit the model (too many degrees of freedom) [18]. We examined graphs with different degrees of freedom to observe their smoothness and whether too many degrees of freedom could potentially compete with the short-term association between variables of interest (Figure S1). We chose seven degrees of freedom per calendar year to allow sufficient flexibility while controlling for seasonality and long-term trends [19] (Figures S1 and S2).

### 2.8. Exposure, Outcome and Confounder

Unadjusted analysis was carried out with diarrhoea count and maximum temperature. Analyses were then adjusted for seasonality and long-term trends using a spline basis with 28 degrees of freedom, i.e., seven knots per year. The model was further adjusted for relative humidity using a two-degrees-of-freedom natural spline in relative humidity (in percentage) to account for nonlinearity, and we used a log-linear term for rainfall. The plots of squared residuals against fitted diarrhoea count showed a nonlinear relationship supporting our use of negative binomial regression [20].

### 2.9. Lagged Exposure

After modelling the immediate effects of maximum temperature on diarrhoea count, we explored whether there might be lagged effects on the outcome [21]. We generated copies of maximum temperature, time-shifted by 0–4 fortnights inclusive and fitted the lagged models. We then re-fitted the maximum temperature-diarrhoea count model at two levels: first a distributed lag model by simultaneously entering all lag terms in one model but acknowledging possible collinearity. To reduce collinearity in the distributed lag model, we applied a simple constraint on the effect estimates for the different lags constraining the effect estimates for lags 1–4 to be the same (a ‘lag-stratified’ distributed lag model). Secondly, we estimated site-stratified lagged effects of maximum temperature on diarrhoea count after adjusting for seasonality and long-term trends, relative humidity and rainfall. Effect estimates are expressed as Incidence Rate Ratio (IRR).

### 2.10. Model-Checking and Sensitivity Analysis

Multiple sensitivity analyses were carried out to check whether the main conclusions were sufficiently robust, including generating deviance residuals and plotting against time (Figure S3); a partial autocorrelation function (PAF) plot of the deviance residuals (Figure S4); and a PAF plot including lagged residuals in the model (Figure S5). Moreover, we varied the degrees of freedom in the spline basis of the time function to allow a change in the amount of control for seasonality and long-term trends (Table S1); exchanged maximum temperature with mean temperature (T-mean) as the main exposure variable; included relative humidity as a linear variable; and modified the lagged terms between lag0 and lag8 fortnights for maximum temperature.

## 3. Results

### 3.1. Descriptive Analysis

**Figure 2:**
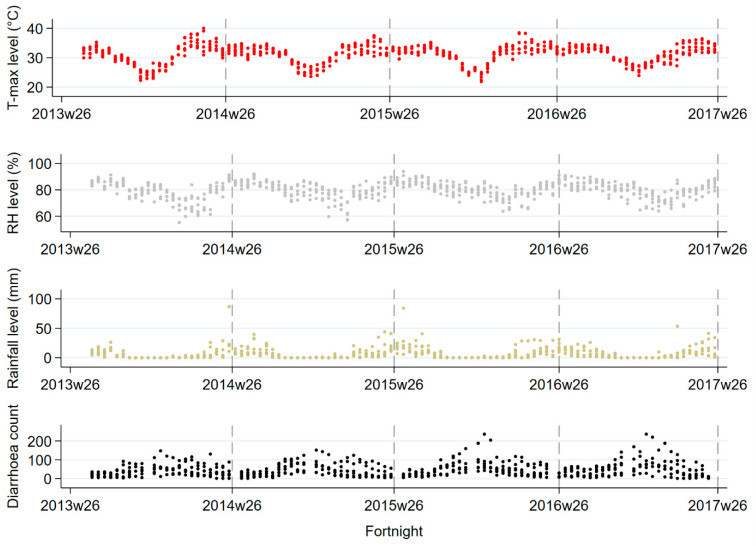
Scatter plots of overall fortnightly regional maximum temperature, relative humidity, rainfall and diarrhoea hospitalisations. T-max—maximum temperature; RH—Relative humidity.

Between August 2013 and June 2017, a total of 25,385 diarrhoea cases were hospitalised. These were in six surveillance hospitals across six administrative divisions. Diarrhoea incidence rates over the study period varied between the highest rate of 695 cases in Barisal and the lowest rate of 44 cases in Chittagong, with an overall rate of 190 cases per 100,000 children under five years old (Table 1). Across six hospitals, the mean diarrhoea count was 46 ± 37 (SD—standard deviation) cases per fortnight. Males predominated, contributing 16,199 cases (63.8%). The median age was 12 months (interquartile range, 8–18) (Table 1). Overall, fortnightly mean (± SD) maximum temperature and relative humidity were 31.1 ± 3.2 °C and 79.7 ± 6.5%, respectively, with a median (range) rainfall of 2.7 (0–86.8) mm (Table 1). Both climate factors and childhood diarrhoea count were dominated by seasonality, as shown in the scatter plots (Figure 2).

### 3.2. Effects of Maximum Temperature

Unadjusted analysis showed a negative effect of maximum temperature on diarrhoea count (Incidence Rate Ratio―IRR per degree C, IRR = 0.937; 95% CI, 0.920–0.955). Analysis adjusted for seasonality and long-term trends showed a positive effect of maximum temperature on diarrhoea count (IRR = 1.046; 95% CI, 1.007–1.088). The analysis further adjusted for relative humidity and rainfall showed a variable effect of maximum temperature on diarrhoea count with increased risks in Barisal (IRR = 1.072; 95% CI, 1.026–1.121) and Sylhet (IRR = 1.056; 95% CI, 1.009–1.106), but a decreased risk in Rajshahi (IRR = 0.941; 95% CI, 0.910–0.973) and Khulna (IRR = 0.940; 95% CI, 0.897–0.985) per 1 °C rise in maximum temperature (Table 2).

### 3.3. Overall Lagged Effects of Maximum Temperature

**Figure 3:**
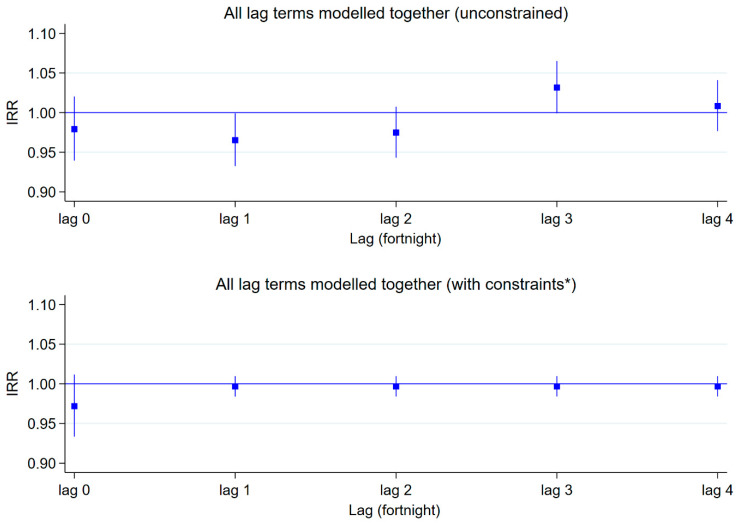
Overall lagged effects of maximum temperature on diarrhoea count, Bangladesh. * The constraint applied was that the lagged associations for fortnights 1–4 were the same.

In the unconstrained distributed lag model, there was an overall decreased risk of diarrhoea per 1 °C rise in maximum temperature at lag 1 (IRR, 0.965; 95% CI, 0.933–0.999) with a marginally increased risk at lag 3 (IRR, 1.032; 95% CI, 1.000–1.065) (Figure 3). In the ‘lag-stratified’ distributed lag model, there was no elevated or reduced risk of diarrhoea at any lag (Figure 3).

### 3.4. Site Stratified Lagged Effects of Maximum Temperature

**Figure 4:**
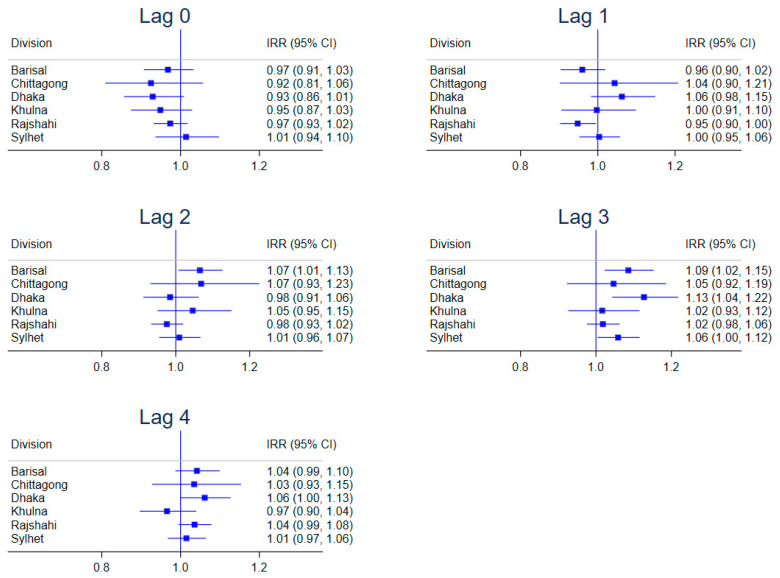
Stratified lagged effects of maximum temperature on diarrhoea count by administrative division, Bangladesh.

In the stratified model, the effects of maximum temperature on diarrhoea incidence varied at different fortnightly lags (Figure 4). A negative association was found at lag 1 in Rajshahi (IRR = 0.948; 95% CI, 0.902–0.995). Conversely, a positive association was found at lag 2 (IRR = 1.065; 95% CI, 1.008–1.127) and lag 3 (IRR = 1.086; 95% CI, 1.023–1.153) in Barisal, at lag 3 (IRR = 1.127; 95% CI, 1.043–1.218) in Dhaka and at lag 3 (IRR = 1.059; 95% CI, 1.004–1.116) in Sylhet with a marginally positive effect estimate at lag 4 in Dhaka (IRR = 1.061; 95% CI, 1.000–1.127).

### 3.5. Effects of Relative Humidity and Rainfall

**Figure 5:**
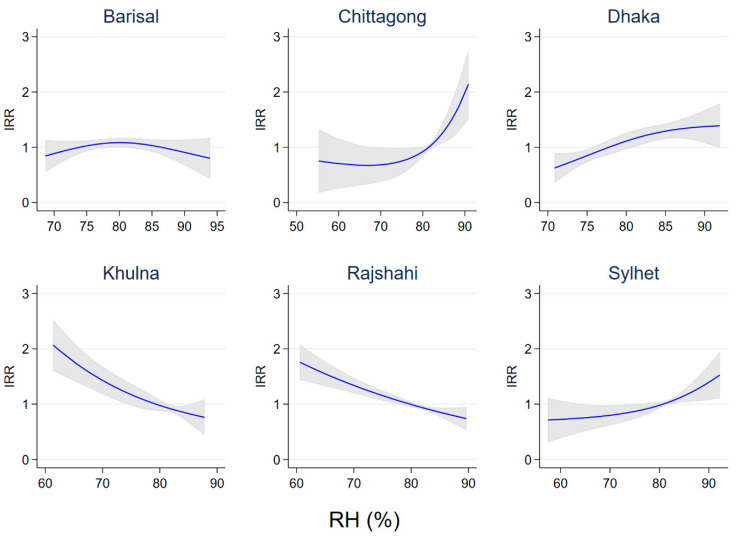
Effects of relative humidity on diarrhoea count by administrative division, Bangladesh.

In the adjusted model, relative humidity showed variable effects on diarrhoea count across the six administrative divisions, with a positive effect in Chittagong, Dhaka and Sylhet, while the effect in Khulna and Rajshahi was negative (Figure 5). Rainfall was associated with a decreased risk of diarrhoea in Chittagong (IRR = 0.524; 95% CI, 0.339–0.810), Dhaka (IRR = 0.626; 95% CI, 0.476–0.822) and Sylhet (IRR = 0.564; 95% CI, 0.438–0.725) for every 10 mm increase in total rainfall (Table 2).

## 4. Discussion

This is the first multi-site time-series study in Bangladesh investigating associations between climate variables and childhood diarrhoea hospitalisations in under five-year-olds. We found that the association between maximum temperature and childhood diarrhoea hospitalisations varied across administrative divisions, suggesting that tailored interventions should be accordingly implemented. In addition, the relationship between maximum temperature and childhood diarrhoea hospitalisations was dominated by seasonality and long-term trends. Stratified lagged effects of maximum temperature on childhood diarrhoea hospitalisations showed spatial heterogeneity in the risk of diarrhoea and that such risks may be delayed by several weeks. The effects of relative humidity on childhood diarrhoea hospitalisations were unequal across administrative divisions. Finally, rainfall showed a negative association with childhood diarrhoea hospitalisations across several administrative divisions.

In our study, we found that only 21% of the total diarrhoea cases were captured by the current surveillance in Chittagong and Dhaka combined, where 60% of children under five years old lived, representing possible suboptimal surveillance coverage in these two divisions. We found nearly two thirds of childhood diarrhoea cases were male. This may in part be explained by the usual male-dominant care-seeking pattern in Bangladesh, meaning that for children such as those in our study, males are more commonly brought to hospitals for admission than female children, as has been shown in other studies for illnesses such as diarrhoea and pneumonia [22,23]. Another reason could be that under-five-year-old males are more commonly affected by diarrhoea than females [24], or possibly because males are genetically more susceptible to infectious diseases, including diarrhoea [24,25]. The median age in our study was slightly higher (12 months vs. 9.5) than in other studies on childhood diarrhoea mortality, with a narrower interquartile range (8–18 months vs. 6–24) [26].

Our study shows that the effects of maximum temperature on childhood diarrhoea hospitalisations are variable and that such effects may be positive or negative. Barisal and Sylhet divisions were the most vulnerable to increased temperature. In Bangladesh and the South Asia region, increased risks of diarrhoea due to high temperatures have been reported in Dhaka, Bangladesh [21], Kathmandu, Nepal [27], Tamil Nadu, India [10] and Bhutan [28]. Although the overall temperature effect in Bangladesh was positively associated with childhood diarrhoea hospitalisations, such effects may be extremely spatially variable across administrative divisions and caution needs to be exercised while interpreting regional and overall effects. In addition to the variable effects of maximum temperature on childhood diarrhoea hospitalisations, the exposure outcome relationship was dominated by seasonality and long-term trends. These results conform with findings from other settings, such as in Jiangsu Province, China, on the prevalence of infectious diarrhoea due to meteorological determinants [29].

One contrasting yet important finding of the maximum temperature-diarrhoea relationship was the negative association in Rajshahi and Khulna. Despite available evidence suggesting a rather complex temperature-diarrhoea association in some European countries [7], stratified analysis in our study enabled us to dismantle, to some extent, complexities in the exposure-response relationship. A recent systematic review suggested that temperature is positively associated with all pathogens, but not viral diarrhoea [30]. Despite a negative maximum temperature-diarrhoea association, we could not rule out that all diarrhoea cases in Rajshahi and Khulna were due to a viral pathogen, which is, however, unlikely to be the case. Both abnormally low and abnormally high temperatures may have a positive association with diarrhoea [31], which further validates our findings that maximum temperature in Bangladesh caused heterogeneous effects on childhood diarrhoea across administrative divisions with different climate and geographical factors.

One key step in this study that further deconstructed the heterogeneity in temperature effects on childhood diarrhoea hospitalisations was the use of stratified lagged effects. Despite the most prominent temperature effects occurring in Barisal and Sylhet, the site-stratified lagged model showed that such effects were not visible until lag 2 in Barisal and lag 3 in Sylhet, with effects offset at lag 4. On the other hand, the negative effects in Rajshahi were only prominent at lag 1. Previous studies have found differences in spatial diarrhoea prevalence in Bangladesh [6]. In our study, variations in temperature effects at different lags not only highlight the spatial heterogeneity in exposure risks, but also underscore how susceptibility to childhood diarrhoea hospitalisations in different regions in Bangladesh may change over time, and how hospitalisations may be delayed by several weeks [29]. This spatiotemporal exposure-lag-outcome phenomenon could potentially make Bangladesh’s diarrhoea prevention efforts challenging, particularly with climate change. Challenges may originate from a failure to recognise temporal risks of diarrhoea with under-reporting and over-reporting contributing to further weakening of an already strained health system, which may have far-reaching consequences of high diarrhoea morbidity and mortality among under-five children.

Spatiotemporal variations in risks of childhood diarrhoea highlight that although climatic conditions, especially temperature, may constitute the most complex determinants in the context of climate change, the myriad factors such as breastfeeding practice, hygienic environment, safe water and sanitation [5,21,32,33] may play a significant role in childhood diarrhoea prevalence in Bangladesh. Studies have been conducted to assess the relationships between behavioural, nutritional and treatment factors, including breastfeeding, hand washing with soap, the prevalence of stunting and wasting, vitamin A and Zinc supplements as well as the use of oral rehydration and treatment for persistent diarrhoea [5]. In addition, strategies including the water, sanitation and hygiene (WASH) program have been well underway for many years, so that WASH is now transitioning from the Millennium Development Goal WASH model to the Sustainable Development Goal WASH model to combat the country’s specific needs around childhood diarrhoea prevention approaches [34]. However, evidence is scarce regarding the roles of climatic factors concerning behavioural and nutritional factors in determining childhood diarrhoea hospitalisations in Bangladesh. The systematic integration of such evidence is advocated by organisations such as UNICEF to promote climate-resilient WASH services in Bangladesh [34]. This study provides a baseline for the preparedness of the Bangladeshi health system in anticipation of childhood diarrhoea epidemics under different climate conditions at different times of the year. Preparedness and response may help with the efficient management of resources by causing less pressure on regional health systems. The risks of diarrhoea due to maximum temperature are heterogeneous; thus, diarrhoea epidemics are likely to be heterogeneous in terms of time and location. The Bangladesh diarrhoea surveillance system may benefit from regional strengthening in line with the recommendation for a strong diarrhoea surveillance system to spatially limit variable diarrhoea incidence at Provincial levels in China [29].

Just as with temperature, relative humidity effects were also unequal across administrative divisions. In contrast to a province in China having had an increased risk of diarrhoea at <80% relative humidity [29], an elevated risk in Chittagong and Sylhet divisions required >80% relative humidity. The present study suggests that Bangladesh administrative divisions are, in general, humid and a non-linear relationship of relative humidity with childhood diarrhoea hospitalisations may cause the exposure-outcome pathways to be more complex. Furthermore, our study suggests that higher humidity may also cause lower childhood diarrhoea incidence, as found in other parts of the world [35]. The unequal effects of relative humidity on childhood diarrhoea incidence could potentially assist with a spatially tailored regional diarrhoea surveillance system in Bangladesh. Recent evidence also favours countries that have a diarrhoea surveillance system that takes into account both etiological and meteorological factors in preventing the spread of infectious diarrhoea [29].

Although the effects of rainfall on childhood diarrhoea incidence were slightly variable across administrative divisions, in general, rainfall was negatively associated with diarrhoea. The strongest negative effect of rainfall was observed in Sylhet. A relatively modest relative humidity effect in Sylhet despite the highest recorded rainfall and a significant temperature effect on childhood diarrhoea incidence supports a negative rainfall-diarrhoea association. This finding is inconsistent with other studies in Bangladesh [21], in the South Asia region [10,27,28] and in the other high-burden regions of the world [35]. We hypothesized one possibility for an overall negative rainfall effect on childhood diarrhoea incidence. Unlike a previous single-site study showing a positive rainfall effect, multi-site analysis in our study estimated stratified effects, where negative effects outweighed positive effects. This type of geographical heterogeneity in rainfall effects has also been supported by studies conducted in other settings [10]. Although heavy rainfall was associated with diarrhoea in many studies, it is proposed that the lagged effects of heavy rainfall on diarrhoea may be location specific and often determined by local transmission dynamics, including the pathogen incubation period for infectious diarrhoea [10].

Our study has some limitations. We did not have data on daily or weekly diarrhoea counts; hence, by using fortnightly intervals, we may have missed shorter-term climate effects on childhood diarrhoea incidence. Our findings may not be entirely representative of urban slums in Dhaka, which is one of the most populous cities in the world. The surveillance hospital for the Dhaka division is located in a district adjacent to Dhaka, not in Dhaka itself, and may not capture childhood diarrhoea cases from its slum dwellers. However, as the climate effects were estimated on spatially related populations in our study, the findings in Dhaka are representative of its slum dwellers.

Other limitations include that diarrhoea surveillance in Bangladesh is conducted in tertiary teaching hospitals, which are referral hospitals, and may not capture the true burden of mild to moderate childhood diarrhoea cases, which are predominantly managed in the outpatient and inpatient departments of subdistrict hospitals. In addition, we were not able to examine the complete picture of diarrhoea epidemiology, as we did not have information on the clinical and biochemical severity of diarrhoea cases or the microbiological assessment of stool specimens. In spite of recent evidence suggesting that rotavirus, *Shigella*, norovirus and adenovirus are widely implicated in causing diarrhoea in children under five years old, we were unable to examine whether these pathogens were affected by climate variability due to the unavailability of data. In an ideal situation, access to stool specimen testing information may help to understand the true burden of bacterial and viral causes of diarrhoea, which would be useful for informing antibiotic prescription patterns for childhood diarrhoea hospitalisations in Bangladesh.

Despite these limitations, the key strength of our study is the generalizability of the findings given the geographical representativeness covering the large administrative divisions in Bangladesh. Our findings are also generalizable to smaller administrative levels in Bangladesh and possibly in other similar settings. In support of this claim, we note that Bangladesh is densely populated, hospitals are overflowing with patients and people do not follow any referral pattern for care seeking, which means a sheer volume of childhood diarrhoea hospitalisations was captured by this surveillance, reflecting population-level burden. In spite of the importance of pathogen information, evidence suggests that a changing climate, particularly rising maximum temperatures, increases the incidence of all-cause and bacterial diarrhoea; hence, our findings are broadly relevant and useful to Bangladesh and other similar countries [30]. In line with recent evidence suggesting targeted interventions for identifying and preventing emerging and re-emerging infections [36], we suggest that Bangladesh health authorities should develop diarrhoea control measures reflecting the climate-sensitive distribution of diarrhoea cases among young children, as found in our study.

## 5. Conclusions

Despite no significant variations in maximum temperature, relative humidity and rainfall across administrative divisions in Bangladesh, substantial variability has been noted in their effects on childhood diarrhoea incidence in different divisions. Such findings are a reminder that a generic national guideline may be inadequate in controlling childhood diarrhoea taking climate change into consideration, and that policymakers should consider regionally tailored diarrhoea prevention strategies. Ideally, the Bangladesh diarrhoea surveillance system needs strengthening to incorporate our findings of spatial and temporal variations in climate effects so as to assist the country in efficiently managing resources for diarrhoea prevention without impacting the health system.

## Figures and Tables

**Table 1:** Meteorological data and characteristics of diarrhoea cases, August 2013–June 2017, by administrative division and overall, Bangladesh.

Division	Total Count	Total Children <5 Years Old *	Incidence Rates ^†^	Male (%)	Age in months—M (IQR)	Count—M ± SD	Maximum Temperature °C—M ± SD	Relative Humidity %—M ± SD	Rainfall mm—M (Range)
Barisal	5997	862,390	695	3821 (63.7)	10 (7–15)	64 ± 25	31.1 ± 2.9	83.3 ± 5.2	2.4 (0–40.8)
Chittagong	1449	3,261,306	44	883 (60.9)	12 (9–18)	16 ± 10	30.6 ± 2.4	77.8 ± 6.8	3.4 (0–86.8)
Dhaka	3847	4,815,057	80	2601 (67.6)	11 (8–15)	42 ± 29	30.1 ± 3.0	81.8 ± 4.3	3.2 (0–33.1)
Khulna	2504	1,403,586	178	1504 (60.1)	13 (9–20)	27 ± 17	32.1 ± 3.6	77.3 ± 6.0	1.7 (0–28.8)
Rajshahi	7593	1,772,061	428	4756 (62.6)	12 (9–18)	82 ± 53	31.8 ± 4.1	79.8 ± 6.7	1.7 (0–18.4)
Sylhet	3995	1,270,094	315	2634 (65.9)	12 (8–17)	43 ± 22	30.9 ± 2.6	78.1 ± 7.3	7.6 (0–53.5)
Overall	25,385	13,384,494	190	16,199 (63.8)	12 (8–18)	46 ± 37	31.1 ± 3.2	79.7 ± 6.5	2.7 (0–86.8)

* Total children only included children 1–4 years old due to unavailability of data—Bangladesh Bureau of Statistics—population census 2011 (the population in each division was considered to be at risk of diarrhoea for the entire study period, and unchanged throughout); ^†^ Incidence rates per 100,000 children < 5 years old; M (IQR)—median (interquartile range); M ± SD—mean ± standard deviation; M (Range)—median (range); Study period—August 2013–June 2017.

**Table 2:** Effect estimates (IRR) of climate variables on diarrhoea count by administrative division, adjusted for covariates *.

Administrative Division	IRR	95% CI	*p* Value
Effects of maximum temperature (per °C)			
Barisal	1.072	1.026–1.121	0.002
Chittagong	1.006	0.940–1.076	0.872
Dhaka	1.011	0.966–1.057	0.645
Khulna	0.940	0.897–0.985	0.009
Rajshahi	0.941	0.910–0.973	0.000
Sylhet	1.056	1.009–1.106	0.020
Effects of rainfall (per 10 mm)			
Barisal	1.128	0.902–1.411	0.292
Chittagong	0.524	0.339–0.810	0.004
Dhaka	0.626	0.476–0.822	0.001
Khulna	1.117	0.793–1.572	0.527
Rajshahi	1.190	0.942–1.505	0.144
Sylhet	0.564	0.438–0.725	0.000

* Covariates included seasonality and long-term trends with relative humidity and rainfall in the maximum temperature-diarrhoea model; and with maximum temperature and relative humidity in the rainfall-diarrhoea model. IRR—incidence rate ratio.

## Data Availability

Restrictions apply to the availability of these data because the data were obtained from a third party. It could be available from the corresponding authors (M.R.R. and P.B.) with the permission of the third party.

## Supplementary Materials

The following supporting information can be downloaded at: https://www.mdpi.com/article/10.3390/ijerph20136279/s1, Figure S1: Modelling seasonality and long-term trends in childhood diarrhoea data, Bangladesh; Figure S2: Residual variation in diarrhoea count after modelling seasonality and long-term trends. * Fitted values were from the time spline model; Figure S3: Scatter plot showing deviance residuals over the study period; Figure S4: Partial autocorrelation function (PAF) plot of the deviance residuals (from adjusted model); Figure S5: Partial autocorrelation function (PAF) plot including 1-fortnight lagged residuals in the model (from autocorrelated model); Table S1: Model checking with different degrees of freedom for the spline basis and their associated Akaike information criteria (AIC) statistics.
