# Supplementary material for: Short-Term Effects of Climate Variability on Childhood Diarrhoea in Bangladesh: Multi-Site Time-Series Regression Analysis
Source: Int J Environ Res Public Health. 2023 Jul 2;20(13):6279. doi: 10.3390/ijerph20136279 (PMC10341980; doi:10.3390/ijerph20136279)
Supplement: Supplementary file 1 [file ijerph-20-06279-s001.zip › ijerph-2463692-supplementary.pdf]

Short-term effects of climate variability on childhood diarrhoea in Bangladesh: multi-site time-series regression analysis

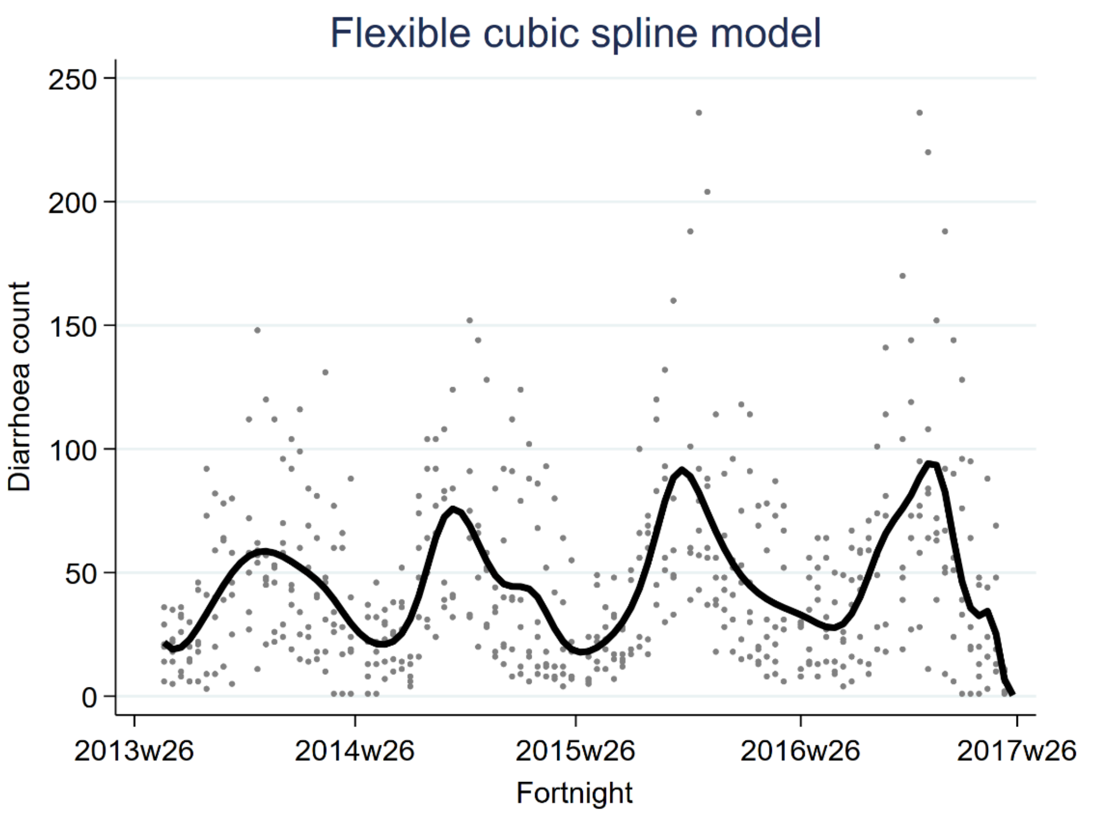

Figure S1. Modelling seasonality and long-term trends in childhood diarrhoea data, Bangladesh.

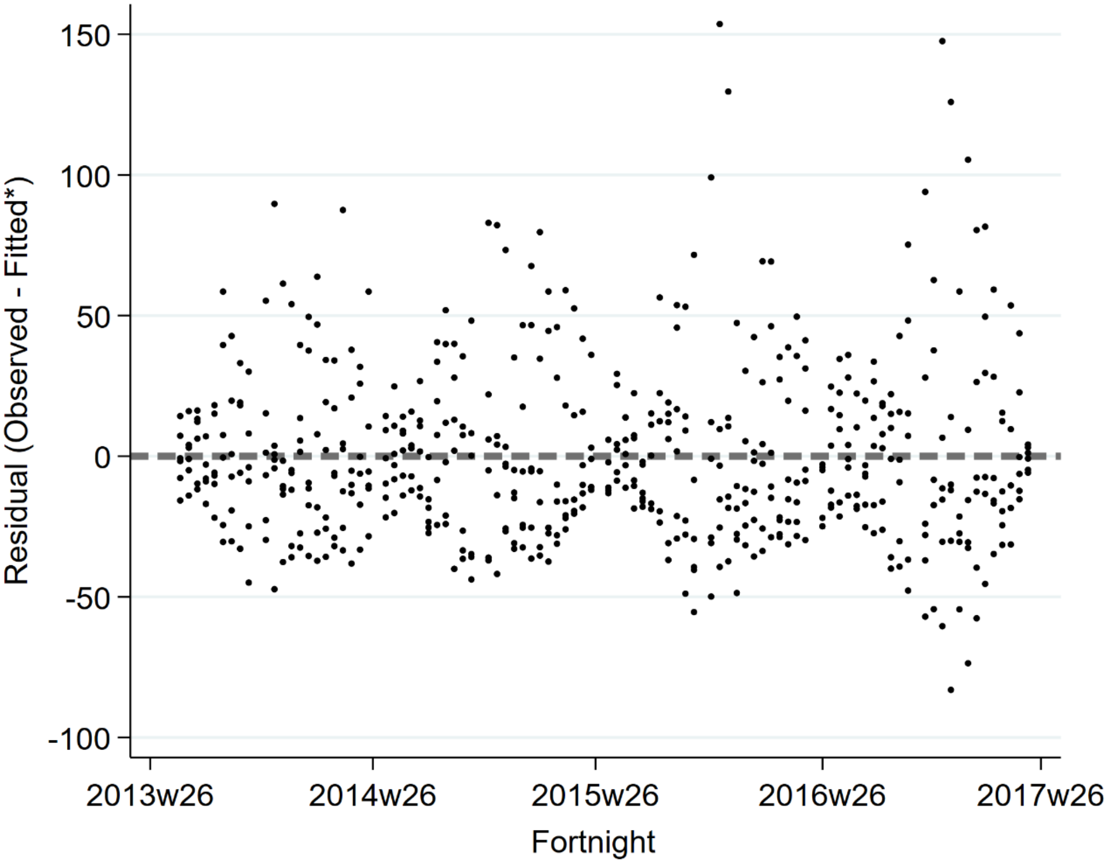

Figure S2. Residual variation in diarrhoea count after modelling seasonality and long-term trends. *Fitted values were from the time spline model.

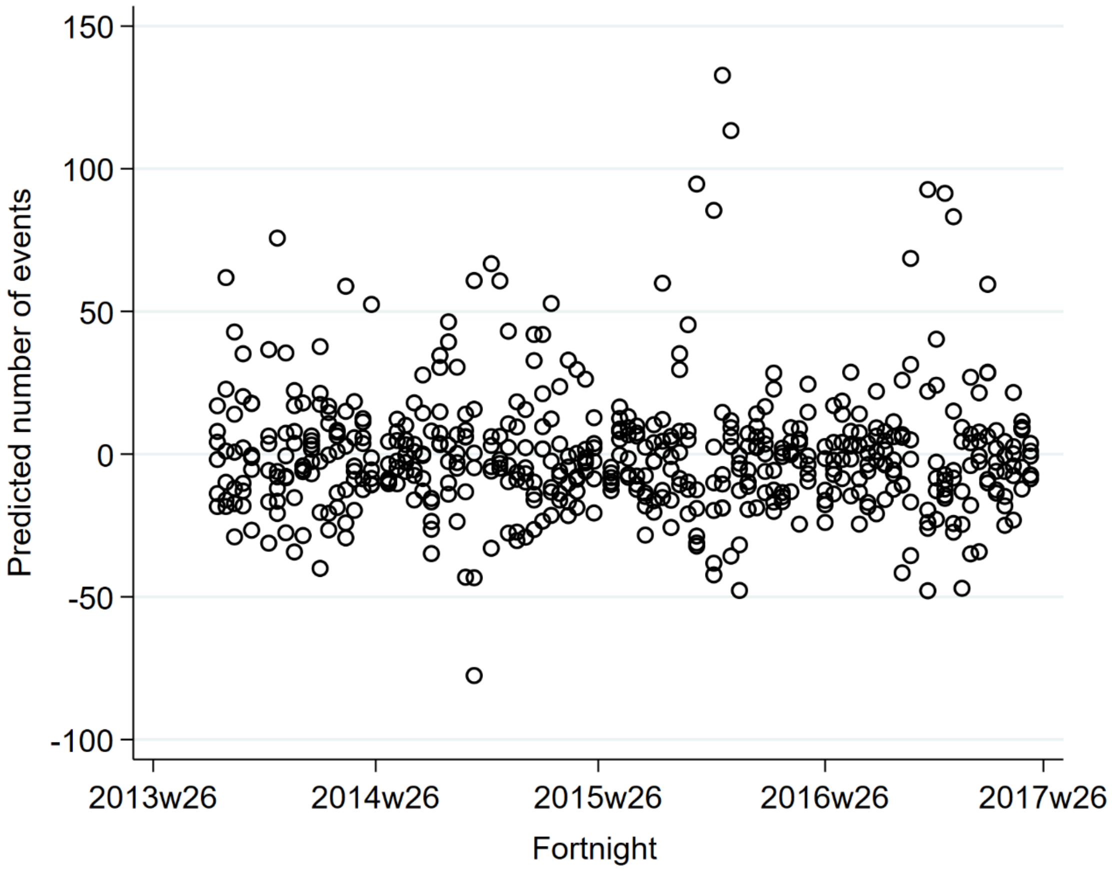

Figure S1. Scatter plot showing deviance residuals over the study period.

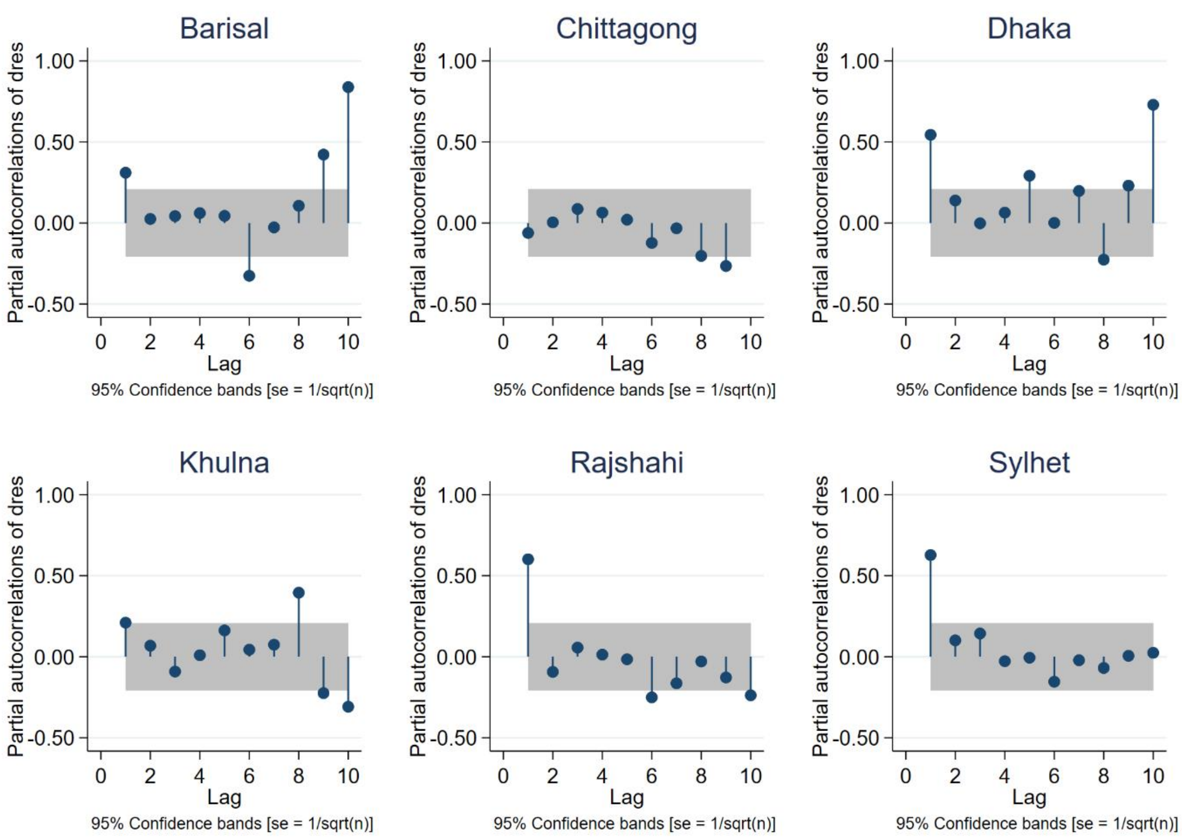

Figure S2. Partial autocorrelation function (PAF) plot of the deviance residuals (from adjusted model).

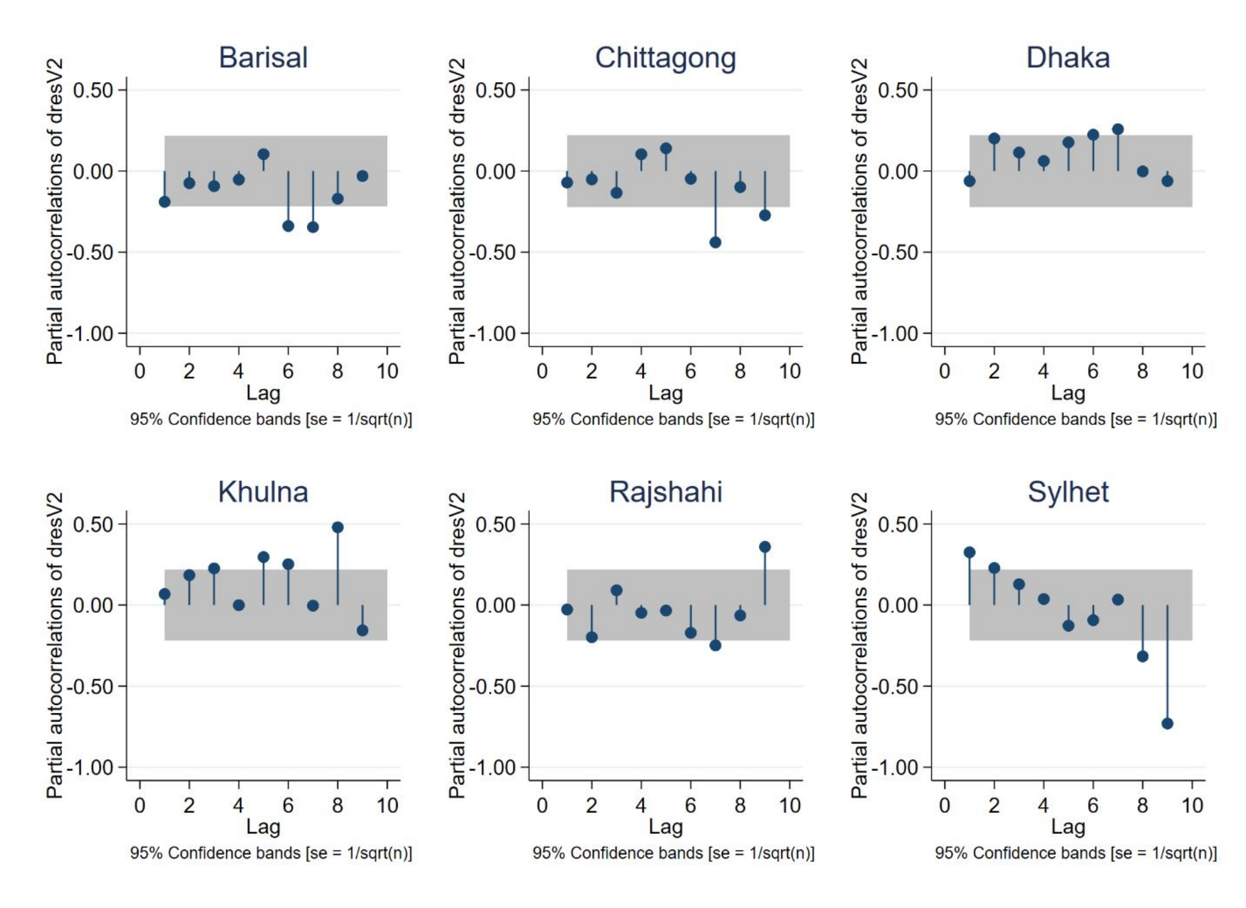

Figure S3. Partial autocorrelation function (PAF) plot including 1-fortnight lagged residuals in the model (from autocorrelated model).

Table S1. Model checking with different degrees of freedom for the spline basis and their associated Akaike information criteria (AIC) statistics.

DF/year	Total DF	Variables in the model	AIC
4	4*4 = 16	T-max, RH, Spline, Diarrhoea count	23.209
5	5*4 = 20	T-max, RH, Spline, Diarrhoea count	22.964
6	6*4 = 24	T-max, RH, Spline, Diarrhoea count	22.817
7	7*4 = 28	T-max, RH, Spline, Diarrhoea count	22.648
